# An Improved Modelling Approach for the Comprehensive Study of Direct Contact Membrane Distillation

**DOI:** 10.3390/membranes11050308

**Published:** 2021-04-22

**Authors:** Abolfazl Ansari, Saman Kavousi, Fernanda Helfer, Graeme Millar, David V. Thiel

**Affiliations:** 1 School of Engineering and Built Environment, Griffith University, Brisbane, QLD 4111, Australia; 2 Independent Researcher, Esteghlal Blvd., Shiraz 71757-43659, Iran; samankavousi@gmail.com; 3 Institute for Future Environments, School of Mechanical, Medical & Process Engineering, Science and Engineering Faculty, Queensland University of Technology (QUT), Brisbane, QLD 4000, Australia; graeme.millar@qut.edu.au

**Keywords:** desalination, membrane distillation, modelling, temperature and concentration polarisation

## Abstract

Direct Contact Membrane Distillation (DCMD) is a promising and feasible technology for water desalination. Most of the models used to simulate DCMD are one-dimensional and/or use a linear function of vapour pressure which relies on experimentally determined parameters. In this study, the model of DCMD using Nusselt correlations was improved by coupling the continuity, momentum, and energy equations to better capture the downstream alteration of flow field properties. A logarithmic function of vapour pressure, which is independent from experiments, was used. This allowed us to analyse DCMD with different membrane properties. The results of our developed model were in good agreement with the DCMD experimental results, with less than 7% deviation. System performance metrics, including water flux, temperature, and concentration polarisation coefficient and thermal efficiency, were analysed by varying inlet feed and permeate temperature, inlet velocity, inlet feed concentration, channel length. In addition, twenty-two commercial membranes were analysed to obtain a real vision on the influence of membrane characteristics on system performance metrics. The results showed that the feed temperature had the most significant effect on water flux and thermal efficiency. The increased feed temperature enhanced the water flux and thermal efficiency; however, it caused more concentration and temperature polarisation. On the other hand, the increased inlet velocity was found to provide increased water flux and reduced temperature and concertation polarisation as well. It was also found that the membrane properties, especially thickness and porosity, can affect the DCMD performance significantly. A two-fold increase of feed temperature increased the water flux and thermal efficiency, 10-fold and 27%, respectively; however, it caused an increase in temperature and concertation polarisation, at 48% and 34%, respectively. By increasing Reynolds number from 80 to 1600, the water flux, CPC, and TPC enhanced by 2.3-fold, 2%, and 21%, respectively. The increased feed concentration from 0 to 250 [g/L] caused a 26% reduction in water flux. To capture the downstream alteration of flow properties, it was shown that the ratio of inlet value to outlet value of system performance metrics decreased significantly throughout the module. Therefore, improvement over the conventional model is undeniable, as the new model can assist in achieving optimal operation conditions.

## 1. Introduction

Water scarcity is increasing globally due to population growth, climate change, and expanded industrial activities, leading to a severe global challenge [1,2,3]. To clear away contaminants and salt from water in various sources, ranging from wastewater to seawater, thermal-based and membrane-based desalination processes have been widely developed [4]. Thermally driven technologies, such as multistage flash distillation, consume high-priced energy to vaporize water. Thus, this method is increasingly being replaced by membrane-based technologies, especially reverse osmosis (RO) [5]. Membrane distillation (MD) is hydrophobic membrane-based desalination technology which is thermally driven [6]. The driving force is the water vapour pressure difference between the feed (hot and salty) and permeate (cold and fresh) streams. MD has several advantages over other desalination processes. First, this method uses low operating temperatures (below 90 °C) which enables the use of waste heat and renewable energy sources [7,8]. Second, the applied pressure in MD is lower compared to the other pressure-driven desalination technology such as reverse osmosis. Thus, mechanical properties are not a major concern in MD. Third, as MD exhibits a high salt removal rate, it can in theory desalinate hypersaline water [9,10].

Not being cost effective in terms of energy efficiency is one of the main MD challenges that should be addressed. One of the main reasons for the low energy efficiency is the heat loss along the module. Meticulously understanding the flow properties alterations along the module is necessary to find a solution for this issue. Several studies have employed semi-empirical Nusselt and Sherwood correlations to investigate DCMD system performance [11,12]. With this approach, the predicted temperatures and water flux are uniform in space. As these correlations only consider one dimensional heat and mass transfer, downstream flow and variables including permeate flux, temperature distribution, concentration and temperature polarisation are unable to be captured. With an emphasis on heat transfer, Phattaranawik et al. [13,14] and Qtaishat et al. [15] analysed heat and mass transfer across a DCMD membrane. Bouchit et al. [16], Manawi et al. [17], and Yang et al. [18] proposed a one-dimensional semi-empirical model to investigate the optimum operating conditions without considering downstream flow alterations. Computational fluid dynamics (CFD) was extensively used as another method to model DCMD. However, CFD suffers from high complexity and consumes excessive time to model the membrane as a porous media. Lou et al. [19,20] conducted CFD simulation to analyze the downstream variation of flow properties. However, they used a linear water flux equation in terms of vapour pressure difference, which is dependent on experimentally determined parameter. Park et al. [21] carried out CFD simulation and experimental studies to investigate the effect of screen spacer on the DCMD process. They reported the insertion of a mesh screen spacer assisted to increase the convective heat transfer. This leads to decreased temperature and concentration polarisation along the membrane module. One aspect of our study was to further develop the one-dimensional semi-empirical model to capture the downstream variables. This allows us to analyse and investigate the importance of localizing heat generation or using direct heated concept on the DCMD performance if the downstream alteration along the module is considerable.

Temperature polarisation and concentration polarisation are two main phenomena that cause a reduction in temperature difference, and consequently in transmembrane vapour flux [22,23,24,25,26] if the operating condition remains constant. Of the studies that have modelled DCMD modules, minimal discussion exists regarding the negative effects of concentration polarisation on DCMD performance [27]. Indeed, most studies focus on temperature polarisation alone. Besides, of the studies that have used CFD to model DCMD [19,21,23,25,28,29,30,31,32,33,34], the majority have not considered solute transport [25,28,29,30,31,32,33,34]; or if they have [21], there is limited discussion on the effects of the concentration polarisation on different parameters of DCMD module performance. 

Although water vapour transmembrane mass flux is the most important parameter in MD system modelling, studies have applied constant fitting parameter, single-gas mass transfer equation, and/or only considered the transition region in their modelling approaches [20,21,35,36]. Yazgan-Birgi et al. carried out a CFD study to compare the flat sheet and hollow fiber DCMD membrane modules in terms of water flux and Temperature Polarisation Coefficient (TPC). Their results indicated that the flat sheet module have 21% higher flux than hollow fiber module. Like Lou et al.’s studies [19,20], they modelled water flux with a linear function of water vapour pressure difference between the hot and cold side of the membrane. However, Dusty Gas Model (DGM), a transport model for simulating the motion of fluid mixtures through a porous media, shows the water flux is a logarithmic function of water vapour pressure. Therefore, another novel aspect of this investigation was to develop a comprehensive study on DCMD module performance, wherein the binary gas mass transport for three different mechanisms, including Knudsen, molecular, and transition regions, have been considered in terms of Knudsen number. 

Membrane characteristics are also critical in MD simulation and MD performance analysis [37,38,39,40,41,42,43,44]. Though most commercial membranes used in MD studies are not marketed as MD, it is important to assess them in terms of pore size, porosity, tortuosity, and thermal conductivity, to enable a thorough study on their performance under DCMD conditions. Limited knowledge exists on the effect of the current commercial membranes on DCMD performance [24,45]. For example, Vanneste et al. [46] analysed 17 commercial membranes in terms of water flux and thermal efficiency [47] without discussing temperature and concentration polarisation. Therefore, another important aspect of this study was to analyse commercially available membranes in terms of their performance for DCMD application.

Within the above context, the following specific objectives were set for this study:

1. To develop and validate a semi-empirical model that is able to capture downstream variables, including transmembrane water flux, Temperature Polarisation Coefficient (TPC), Concentration Polarisation Coefficient (CPC), and thermal efficiency on a DCMD module;
2. To model DCMD with a self-sustained water flux equation which allows to systematically investigate different membranes with few assumptions;
3. To conduct a sensitivity analysis to understand which parameters have more significant effects on DCMD performance;
4. To analyse 22 commercially available membranes in terms of performance metric evaluation to assess the suitability of these membranes for DCMD application.

The methodology involved the use of computational modelling wherein the channel and membrane were sub-divided into *n* elements on the basis that transmembrane mass and heat transfer, and flow through the channels, were directly dependent on temperature alterations along the membrane and channel. Employing continuity, Navier-Stokes, and energy equations, *n* feed, and permeate bulk temperatures along the channel were calculated. Based on the feed and permeate bulk temperatures, and Nusselt correlation, the temperature on both sides of the membrane, permeate flux, and all system performance metrics, including TPC and CPC, and thermal efficiency were derived.

## 2. Governing Equations

In general, MD can be conveniently defined into three stages: feed-side, membrane, and permeate-side. The process happening in each stage leads to a resistance to heat and mass transfer. 

### 2.1. Transmembrane Transport

#### 2.1.1. Transmembrane Mass Transport

As in MD, isothermal conditions are assumed. Based on the dusty gas model [48], the water vapour transport inside the pores of the membrane can be explained in three mechanisms: viscous flow, Knudsen flow, and Continuum diffusion [48]. The combination of these mechanisms can better describe the exact water vapour transport. Stephan-Maxwell equations describe the diffusive flows of multicomponent mixtures. Fick’s law, a special case of Stephan-Maxwell equations, and Darcy’s law can be employed to describe the binary mixture diffusive flow and viscous flow, respectively [48].

Two main mass transport assumptions were made to study gas transport through the membrane as a porous media: single gas and binary gas mixture through porous media. Two main gases are involved in the membrane: air and water vapour. The binary gas mixture is considered to have more exact mass transport model through membrane. The value of Knudsen number needs to be calculated to select the mechanism applicable to model the mass transport through the membrane (Appendix A Equations (A1) and (A2)).

The water mass flux (J) can be described through three mechanisms as a function of the Knudsen number (Equations (1)–(4)). When kn>1, the free molecular region, the mass flux is described by the Knudsen mass transfer Equation (2). In the case of kn<0.01, with the assumption of having uniform pressure in the membrane pores, the water mass flux is calculated by Equation (3). In the case of 0.01<kn<1, the water vapour diffusive flux is obtained by Equation (4) [48]:

(1) $$J=\{\begin{array}{l} J_{Kn}\;,\;\;\;\;\;\;kn>1\\ J_{tran\;,\;\;\;\;\;\;}0.01<kn<1\\ J_{mol}\;,\;\;\;\;\;\;kn<0.01 \end{array}$$

(2) $$J_{Kn}=\frac{M_{w}}{RT}\cdot \frac{2\epsilon d_{p}}{3\delta \tau }{\left[\frac{2RT}{\pi M_{w}}\right]}^{0.5}\left(p_{v,fm}-p_{v,pm}\right)$$

(3) $$J_{mol}=\frac{\left(\epsilon /\tau \right)P_{t}D_{v-a}M_{w}}{\left(1-\alpha \right)\delta RT}ln\left[\frac{P_{t}-\left(1-\alpha \right)p_{v,pm}}{P_{t}-\left(1-\alpha \right)p_{v,fm}}\right]$$

(4) $$J_{tran}=\frac{\left(\epsilon /\tau \right)P_{t}D_{v-a}M_{w}}{\left(1-\alpha \right)\delta RT}ln\left[\frac{D_{kn}\left[P_{t}-\left(1-\alpha \right)p_{v,pm}\right]+\left(\epsilon /\tau \right)P_{t}D_{v-a}}{D_{kn}\left[P_{t}-\left(1-\alpha \right)p_{v,fm}\right]+\left(\epsilon /\tau \right)P_{t}D_{v-a}}\right]$$

where J is the mass flux of water, $J_{Kn}$ is the mass flux of water in the case of Knudsen region, $\;J_{mol}$ is the mass flux in the case of molecular region, $J_{tran}$ is the mass flux in the case of transition region, $M_{w}$ is the molecular weight of water, $M_{a}$ is the molecular weight of air, α is the ratio of molecular weight, R is the universal gas constant, ϵ is the membrane porosity, $d_{p}$ is the membrane pore diameter, δ is the membrane thickness, τ is the membrane tortuosity, $P_{t}$ is the total pressure in the membrane pores, $D_{kn}$ is the Knudsen diffusion coefficient, $D_{v-a}$ is the water-vapour diffusivity in the air, T is the membrane temperature, $p_{v,fm}$ is the water vapour pressure on the feed side of membrane, and $p_{v,pm}$ is the water vapour pressure on the permeate side of membrane.

#### 2.1.2. Transmembrane Heat Transfer

**Figure 1:**
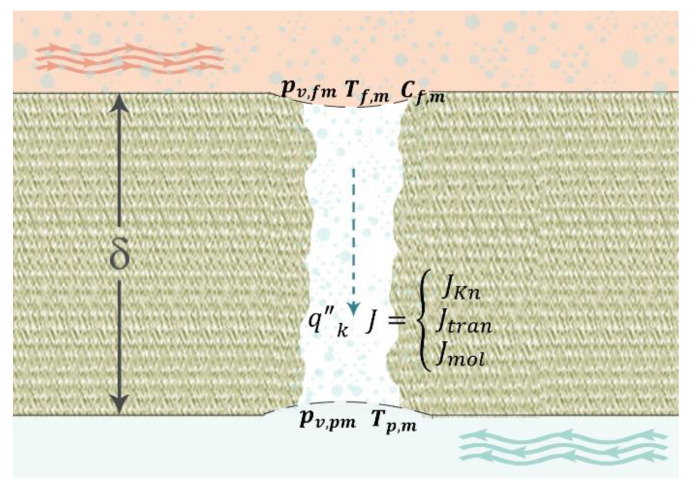
Sketch (not to scale) illustrating mass transfer and conduction heat transfer through a MD membrane. Temperature and water vapour pressure differences are the driving forces across the hydrophobic membrane leading to water vapour and heat transfers.

Two contributions to the heat transfer in the membrane exist. First, the heat conduction transfer through the membrane and, second, the latent heat transfer owing to the flux of water vapour. As illustrated in Figure 1, the conduction heat transfer through the membrane can be modelled using Equations (5)–(7).

(5) $${q^{\prime \prime }}_{k}=h_{m}\left(T_{f,m}-T_{p,m}\right)$$

(6) $$h_{m}=\frac{\kappa_{e}}{\delta }$$

(7) $${q^{\prime \prime }}_{fg}=Jh_{fg}$$

where ${q^{\prime \prime }}_{k}$ is the transmembrane conductive heat transfer flux, $h_{m}$ is the membrane heat transfer coefficient, $T_{f,m}$ is the membrane feed side temperature, $T_{p,m}$ is the membrane permeate side temperature, $\kappa_{e}$ is the effective thermal conductivity of membrane, δ is the membrane thickness, and $h_{fg}$ is the latent heat. 

In the literature, mainly two models have been employed to predict the effective thermal conductivity of membrane—the parallel model and series model. As the membrane is comprised of polymer, water vapour, and air, to predict the thermal conductivity of membrane, all components must be considered. The thermal conductivity of trapped air and water vapour is similar, and both can be estimated by Equations (A9) and (A10) [49].

### 2.2. Channel Flow Governing Equations

The semi-empirical approximation using Nusselt correlation and numerical simulation are two approaches which are used to calculate the temperature along the membrane.

#### 2.2.1. Semi-Empirical Approximation Using Nusselt Correlation

The feed and permeate heat transfer coefficients can be calculated using dimensionless Nusselt number, which can be obtained using semi-empirical correlations (Equations (A13)–(A20)) [50].

To calculate the feed and permeate temperatures at the membrane surface and, accordingly, calculate temperature polarisation as one of the main causes of the loss of driving force in MD, heat transfer balance must be applied by Equation (8).

(8) $$\begin{array}{c} {Q^{\prime \prime }}_{feed}={Q^{\prime \prime }}_{membrane}={Q^{\prime \prime }}_{Permeate}\\ -k_{f}{\frac{\partial T}{\partial y}|}_{y=0^{+}}=Jh_{fg}-\frac{\kappa_{e}}{\delta }\left(T_{f,m}-T_{p,m}\right)=-k_{f}{\frac{\partial T}{\partial y}|}_{y=0^{-}} \end{array}$$

where ${Q^{\prime \prime }}_{feed}$,$\;{Q^{\prime \prime }}_{membrane}$, and $\;{Q^{\prime \prime }}_{Permeate}$ are heat flux in feed channel, heat flux in membrane, and heat flux in permeate channel, respectively.

As for Equation (8), in MD, the heat flux in the membrane, feed, and permeate must be equal. We estimate the feed and permeate heat transfer with the Nusselt empirical Equations (A21) and (A22).

#### 2.2.2. Numerical Method

The governing equations for the laminar flow in the feed and permeate channel are the continuity, Navier-Stokes, and energy equations, as follows:

(9) $$\nabla \cdot \vec{V}=0$$

(10) $$\rho \left[\frac{\partial \vec{V}}{\partial t}+\left(\vec{V}\cdot \nabla \right)\vec{V}\right]=-\nabla p+\mu \nabla^{2}\vec{V}$$

(11) $$\frac{\partial T}{\partial t}+\left(\vec{V}\cdot \nabla \right)T=\frac{k_{f}}{\rho c_{p}}\nabla^{2}T$$

where $\vec{V}=\left[u\;v\right]$ is the fluid velocity vector, T is the temperature, p is the pressure, μ is the viscosity,$\;k_{f}$ is the thermal conductivity, $c_{p}$ is the fluid heat capacity, and ρ is the mixture density.

## 3. Materials and Methods

### 3.1. Process Modelling

**Figure 2:**
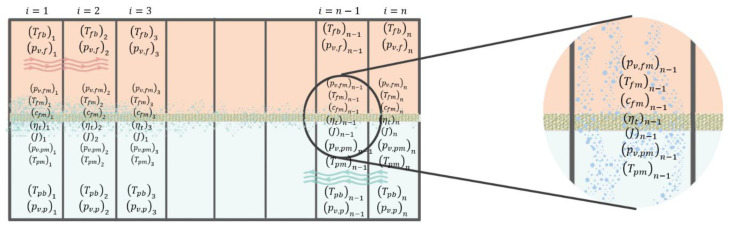
Elements used to study the downstream properties of a DCMD module. “T”, “c”, and “$p_{v}$’ represent the temperature, salt concentration, and water vapour pressure, respectively. Subscripts “f” and “p” refer to the feed and permeate sides, respectively, and ‘b’ and ‘m’ to bulk and membrane locations in the module.

**Figure 3:**
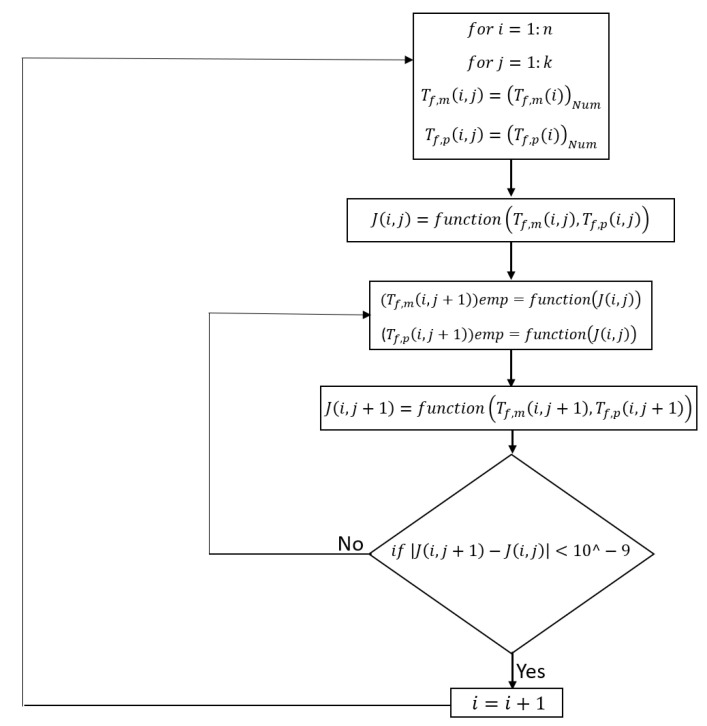
Flowchart illustrating the algorithm developed to include the effect of water vapour mass flux in the calculation of membrane temperature. n and k are the number of elements and iterations.

As shown in Figure 2, the feed and permeate channels are divided into *n* elements, and the Navier-Stokes, continuity, and heat transfer equations are solved by employing Fluent ANSYS. As the heat along the feed channel transfers to the permeate side, the bulk feed temperature along the membrane decreases, whereas the permeate temperature increases. The obtained bulk temperatures enable us to calculate downstream flow properties and membrane temperatures on both sides. Since the equations to compute membrane temperature on both sides in the semi-empirical model are dependent on water flux, and to include the effects of water flux on the temperature results on the numerical model, the algorithm illustrated in Figure 3 has been applied to couple the temperature results of numerical simulation with the empirical model for each element.

The pressure-based method, the SIMPLE algorithm, and a second-order accuracy for the spatial discretization scheme were employed to solve the steady state governing equations. The convergence criterion was defined to $10^{-12}$ for continuity, velocity, and energy equations’ residuals. Velocity-inlet and pressure outlet boundary conditions were set at feed and permeate inlets and outlets, respectively. Wall and coupled boundary conditions were used at the upper and lower walls, and at the interface, respectively [51,52].

**Figure 4:**
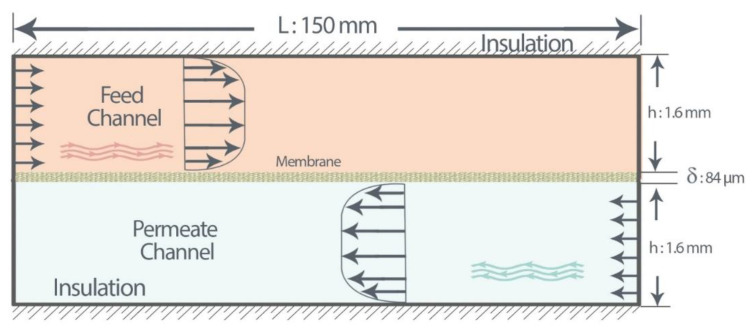
Schematic of flow configuration, counter-current operation, as a baseline for DCMD system. L, length of the channels; h, height of the feed and permeate channel; δ, membrane thickness.

To validate our model with experiment data [21], as illustrated in Figure 4, a two-dimensional flat-sheet membrane module with channel length of *L* and height of *h* as a baseline system, consisting of a PTFE membrane, was considered. The porosity, tortuosity, pore diameter, and thickness of the membrane are 0.62, 2.34, 0.36 µm, and 84 µm, respectively [21]. However, to examine membrane characteristics, we modeled all the 22 commercial membranes discussed in Section 4.6.

A comprehensive parametric study on DCMD system was undertaken. As presented in Table 1, the effect of inlet feed and permeate temperature, inlet velocity, channel length, feed concentration, and membrane characteristics for counter-current flow configuration with regards to water flux, CPC, TPC, and thermal efficiency on DCMD system was explored. 

### 3.2. System Performance Metrics

Some metrics evaluations were used to assess the performance of MD, including TPC, CPC, water flux, and thermal efficiency. 

#### 3.2.1. Temperature Polarisation Coefficient (TPC)

To assess the convective heat flux in the flow channels and the effect of temperature boundary layer and temperature polarisation, the TPC was calculated. The TPC is defined as the ratio of the temperature difference across the membrane surfaces to the temperature difference between the bulk feed and the bulk permeate, as follows:

(12) $$TPC=\frac{T_{f,m}\left(x\right)-T_{p,m}\left(x\right)}{T_{f,b}-T_{p,b}}$$

where $T_{f,m}\left(x\right)$ and $T_{p,m}\left(x\right)$, represent the feed and permeate membrane temperature, $T_{f,b}$ and $T_{p,b}$ are the bulk feed and permeate temperature, respectively. TPC = 1 indicates no temperature polarisation and high convective heat flux, and TPC = 0 indicates high polarisation effect.

#### 3.2.2. Concentration Polarisation Coefficient (CPC)

To characterise concentration polarisation and measure lateral mass concentration in the feed-side channel, CPC is calculated, as follows:

(13) $$CPC=\frac{C_{f,m}\left(x\right)}{C_{f,b}\left(x\right)}$$

where $C_{f,m}\left(x\right)$ and $C_{f,b}\left(x\right)$ represents the concentration on the surface of the membrane feed-side and bulk feed fluid, respectively. CPC = 1 indicates no concentration polarisation and as CPC increases, the concentration polarisation effect increases.

#### 3.2.3. Thermal Efficiency

Thermal efficiency defines the ratio of latent heat of evaporation to the total heat transfer across the membrane, as follows:

(14) $$\eta_{t}=\frac{{q^{\prime \prime }}_{fg}}{{q^{\prime \prime }}_{fg}+{q^{\prime \prime }}_{k}}$$

where ${q^{\prime \prime }}_{k}$, ${q^{\prime \prime }}_{fg}$, and $\eta_{t}$ are the conduction heat transfer flux, phase change heat transfer flux, and thermal efficiency, respectively.

## 4. Results and Discussion

### 4.1. Model Validation

**Figure 5:**
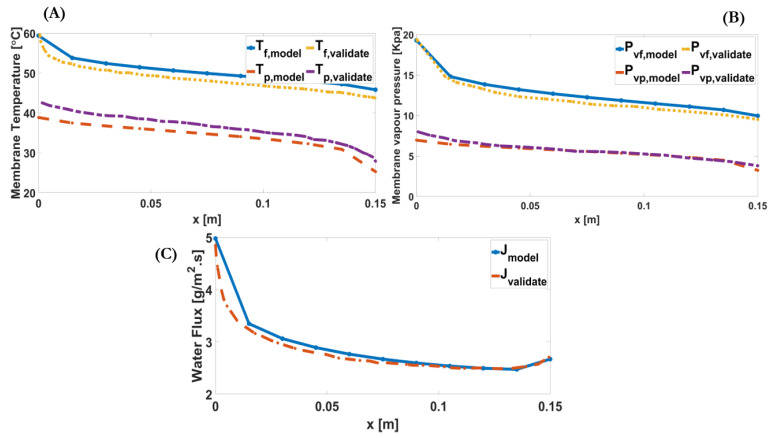
(**A**) Downstream alterations of feed and permeate membrane temperatures validated by the reference model [21]. (**B**) Downstream alterations of the membrane water vapour pressures of the feed and permeate validated by the reference model [21]. (**C**) Downstream alterations of the transmembrane mass flux validated by the reference model (less than 7% deviation) [21].

Downstream alterations of the water flux, membrane temperatures, and membrane vapour pressures along the module were compared with the results of reference [21] for validation, as shown in Figure 5, which were in good agreement, with less than 7% deviation. 

Four level of meshes have been generated to assess the independency of discretized mesh on the solution. The 80×100 mesh seems to be the most efficient compared to the other meshes. Mesh grid study, temperature, and velocity contour for the baseline condition as well as membrane temperature and water vapour pressure difference for different parameters are provided in the Supplementary Materials (Figures S1–S8).

**Figure 6:**
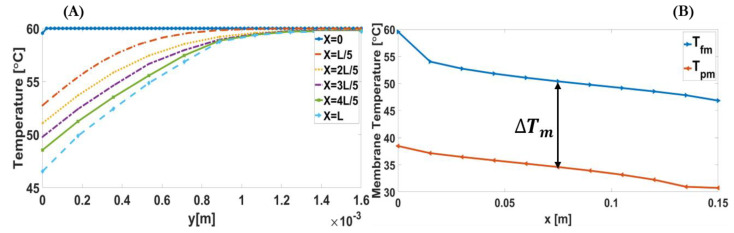
(**A**) Temperature profiles at x=0, x=L/5, x=2L/5, x=3L/5, x=4L/5, and x=L, illustrating thermal boundary layer in the feed channel. (**B**). Alterations of feed and permeate membrane temperatures, and temperature difference of the baseline condition in counter-current operation of DCMD system.

As illustrated in Figure 6A, cross-sectional temperature profiles along the feed channel have been used to show the temperature distribution in each feed side section. It is illustrated that the thermal boundary layer increases and expands along the module; therefore, the heat transfers gradually across the channels on both sides (feed and permeate). Figure 6B shows the decrease in membrane feed temperature in its downstream direction, especially close to the channel inlet, and the increase in membrane permeate temperature in the upstream direction. It also shows that the temperature difference, as the main driving force, becomes almost unchanged after the feed and permeate channel inlets. In conclusion, the results illustrate that contrary to one-dimensional conventional model, temperature profiles in each section and membrane temperatures at both sides (feed and permeate) change significantly along the module. 

### 4.2. Influence of Inlet Feed and Permeate Temperature

To study the influence of inlet feed and permeate temperature, a counter-current arrangement of a DCMD system was modelled for the inlet feed and permeate temperatures $40\leq T_{f}\leq 80$ °C and $20\leq T_{p}\leq 40$ °C, respectively. All other parameters were maintained constant as the baseline condition (Table 1).

**Figure 7:**
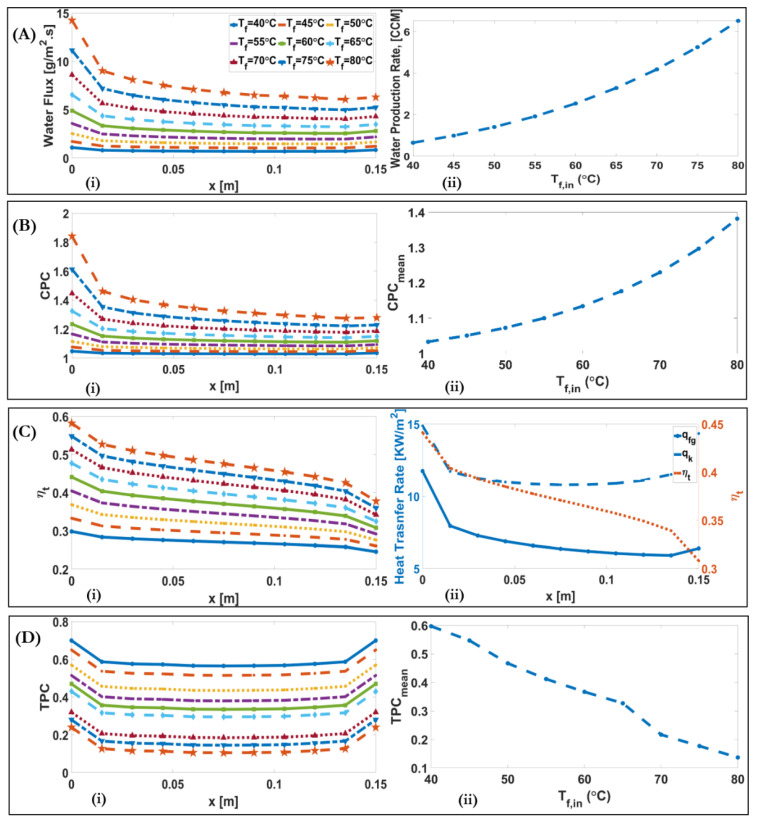
(**A**) (**i**) Downstream alterations of water flux for different inlet feed temperatures of a DCMD system. (**A**) (**ii**)**.** Alteration of water production rate with varying inlet feed temperatures of a DCMD system; (**B**) (**i**) Downstream alterations of CPC for different inlet feed temperatures of a DCMD system. (**B**) (**ii**) Alteration of maximum CPC with varying inlet feed temperatures of a DCMD system; (**C**) (**i**) Downstream alterations of $\eta_{t}$ for different inlet feed temperatures of a DCMD system. (**C**) (**ii**) Conduction heat transfer (${q^{\prime \prime }}_{k}$), phase change (${q^{\prime \prime }}_{fg}$), and thermal efficiency ($\eta_{t}$) of a DCMD system for the baseline condition; (**D**) (**i**) Downstream alteration of TPC for different inlet feed temperatures of a DCMD system, (**D**) (**ii**) Alteration of mean value of TPC with varying inlet feed temperatures.

**Figure 8:**
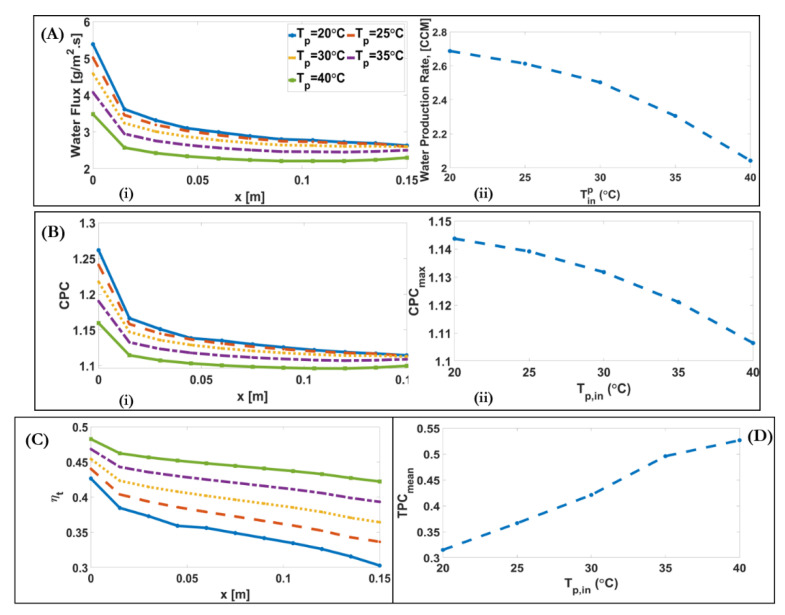
(**A**) (**i**) Downstream alterations of water flux for different inlet permeate temperature of a DCMD system; (**A**) (**ii**) Alteration of water production rate with varying inlet permeates temperatures of a DCMD system. (**B**) (**i**) Downstream alterations of CPC for different inlet permeate temperatures of a DCMD system; (**B**) (**ii**) Alteration of maximum CPC with varying inlet permeate temperatures of a DCMD system. (**C**) Downstream alteration of $\eta_{t}$ for different inlet permeate temperatures of a DCMD system. (**D**) Alteration of mean value of TPC with varying inlet permeate temperatures.

Figure 7A and Figure 8A show the effect of inlet feed and permeate temperature on the transmembrane water flux. Water flux, J, is a logarithmic function of vapour pressure, $p_{v}$, in the case of molecular diffusion and transition regions (our case), and vapour pressure itself is an exponential function of temperature. Therefore, as the membrane temperature difference along the module increases, J increases in an exponential manner. Figure 7A(ii) and Figure 8A(ii) show that with the two-fold increase of feed temperature from 40 °C to the 80 °C, and permeate temperature from 20 °C to the 40 °C, the water production rate increases tenfold, from 0.65 to 6.5 CCM, and decreases from 2.7 to 2 CCM, respectively. Figure 7A(ii) and Figure 8A(ii) illustrate that, as the temperature difference between feed and permeate increases, the downstream alteration of water flux, the ratio of inlet value to outlet value, along the module increases, from 1.3 for the case of $T_{f}=40\;{}^{\circ}C$, $T_{p}=25\;{}^{\circ}C$, to 2.3 for the case of $T_{f}=80\;{}^{\circ}C$, $T_{p}=25\;{}^{\circ}C$. This result shows although increasing temperature difference leads to higher water flux, it needs to find a way to lessen the decrease of water flux along the module, such as localizing heat transfer.

Figure 7B and Figure 8B show the effect of inlet feed and permeate temperatures on CPC. CPC is an exponential function of J and $K_{s}{}^{-1}$. The $CPC_{max}=1.82$ occurs in the case of $T_{f}=80\;{}^{\circ}C$, $T_{p}=25\;{}^{\circ}C$. Figure 7B(ii) and Figure 8B(ii) illustrate that, by increasing $T_{f}$, from 40 to 80 °C, CPC is increased by 34%, and by increasing $T_{p}$ from 20 to 40 °C, CPC decreases by 3%. CPC decreases along the module due to the decreased water flux.

Figure 7C and Figure 8C show the effect of inlet feed and permeate temperatures on thermal efficiency and heat transfer across the membrane. The results show that the thermal efficiency significantly decreases throughout the membrane, reaching 0.75 times the thermal efficiency at the inlet, in the case of $T_{f}=80\;{}^{\circ}C$, $T_{p}=25\;{}^{\circ}C$. Figure 7C(ii) shows the downstream alteration of conduction heat transfer (${q^{\prime \prime }}_{k}$), and phase change heat transfer (${q^{\prime \prime }}_{fg}$), and thermal efficiency ($\eta_{t}$) on the baseline condition. As the conduction heat transfer across the membrane is dependent on the temperature difference between the feed and permeate, the conduction heat transfer decreases, and then at the feed channel outlet and the permeate channel inlet it increases in a rightward direction, as shown in Figure 7C(ii). 

Figure 7D and Figure 8D illustrate the effect of inlet feed and permeate temperatures on the TPC. It is found that the TPC decreases approximately 0.8 times of TPC at the inlet, and then inversely increases to the initial value at the exit. As shown, TPC decreases with the increase of $T_{f}$ from 0.6 for $T_{f}=40\;{}^{\circ}C$ to 0.12 for $T_{f}=80\;{}^{\circ}C$, and increases with the increase of $T_{p}$ from 0.32 for $T_{p}=20\;{}^{\circ}C$ to 0.52 for $T_{p}=40\;{}^{\circ}C$. With the increase of $T_{f}=40\;{}^{\circ}C$ to $T_{f}=80\;{}^{\circ}C$, the $T_{f,b}-T_{p,b}\;$and J becomes 3.6-fold and 10-fold, respectively. If Equation (A21) is divided to $T_{f,b}-T_{p,b}\;$, the ratio of increased J is higher than the increased feed bulk temperature. Therefore, the ratio of total thermal resistance of membrane to the total thermal resistance of the channels and membrane decreases. 

### 4.3. Influence of Inlet Velocity

To examine the effects of the inlet velocity, a counter-current arrangement of a DCMD system was modelled for 0.01$\leq V_{in}\leq 0.2\;m/s$, corresponding to the laminar Reynolds numbers of 80≤Re≤1600  while all other parameters were maintained as the baseline condition.

**Figure 9:**
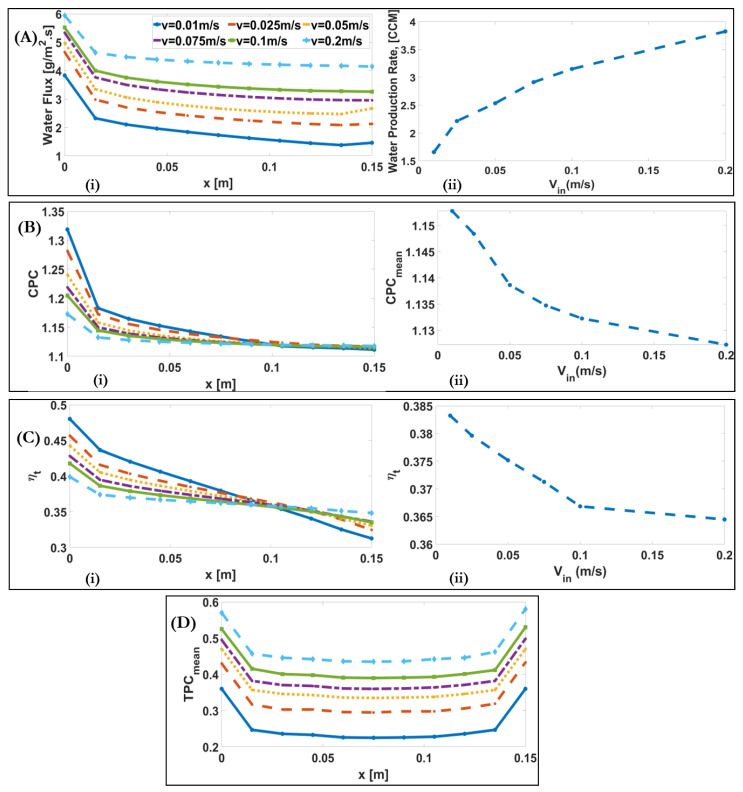
(**A**) (**i**) Downstream alterations of water flux for different inlet velocities of a DCMD system; (**A**) (**ii**) Alteration of water production rate with varying inlet velocities of a DCMD system. (**B**) (**i**) Downstream alterations of CPC for different inlet velocities of a DCMD system; (**B**) (**ii**) Alteration of maximum CPC with varying inlet velocities of a DCMD system. (**C**) (**i**) Downstream alteration of $\eta_{t}$ for different inlet velocities of a DCMD system; (**C**) (**ii**) Alteration of mean $\eta_{t}$ with varying inlet velocities of a DCMD system. (**D**) Downstream alteration of TPC for different inlet velocities of a DCMD system.

Figure 9A(i) shows that, as the inlet velocity increases, the ratio of inlet value to the outlet value of J, the alteration of water flux along the membrane, decreases, from 2.5 for $V_{in}=0.01\;m/s$ to 1.43 for $V_{in}=0.2\;m/s$. J increases approximately 2.3-fold with increasing $V_{in}$. This is because the increased velocity increases turbulence, and consequently the thermal boundary layer becomes thinner, which reduces the temperature polarisation effect, yielding a higher water production rate. 

Figure 9B–D show the downstream alteration of CPC, thermal efficiency, and TPC for different inlet velocities. It is illustrated that, although J increases the CPC slightly decreases from 1.15 for $V_{in}=0.01\;m/s$ to 1.127 for $V_{in}=0.2\;m/s$. This is because two opposite factors affect the CPC. On the one hand, the increase of water flux causes the increase in CPC. On the other hand, the increase in solute convection mass transfer coefficient, and concentration difference between the feed and membrane leads to decrease in CPC. For example, at the inlet, J and $K_{s}$ increase from 3 $g/m^{2}s$ and $1.43\times 10^{-5}\;m/s$ for $V_{in}=0.01\;m/s$ to 6 $g/m^{2}s$ and $3.83\times 10^{-5}\;m/s$ for $V_{in}=0.2\;m/s$, respectively. As illustrated in Figure 9B(i), at the inlet the effect of water flux on the CPC is higher than the convection mass transfer for lower velocities. It was shown that $\eta_{t}\;$ slightly decreases in Figure 9C. This is because ${q^{\prime \prime }}_{fg}$ and ${q^{\prime \prime }}_{k}$ both increase with the increase of $V_{in}$. The increase in ${q^{\prime \prime }}_{k}$ and ${q^{\prime \prime }}_{fg}$ are due to the decrease in temperature polarization effect and the increase in water flux, respectively. TPC decreases approximately 0.7 times of the TPC at the inlet, and then gradually increases. By increasing the inlet velocity from 0.01 to 0.2 m/s, the turbulence increases, and consequently the thermal boundary layer becomes thinner; therefore, the effect of temperature polarisation decreases, and, as a result, TPC increases from 0.26 for $V_{in}=0.01\;m/s$ to 0.47 for $V_{in}=0.2\;m/s$. 

### 4.4. Influence of Inlet Feed Concentration 

To examine the influence of inlet feed concentration, a counter-current arrangement of a DCMD system was modelled for the inlet feed concentration $0\leq C_{f}\leq 250\;g/L$ and all other parameters were maintained as the baseline condition.

**Figure 10:**
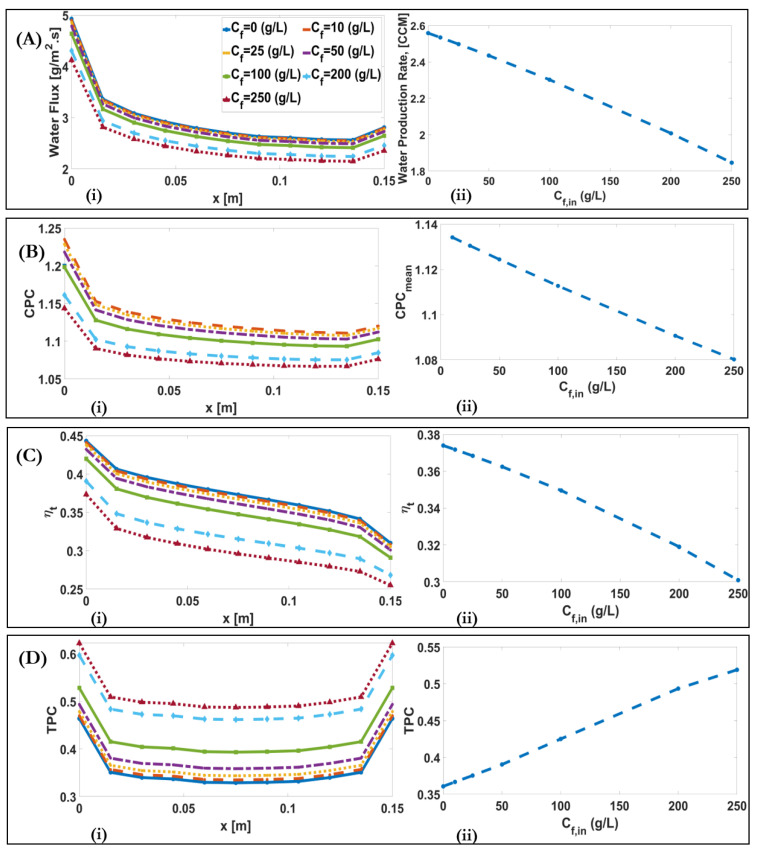
(**A**) (**i**) Downstream alterations of water flux for different inlet feed concentrations of a DCMD system; (**A**) (**ii**) Alteration of water production rate with varying inlet feed concentrations of a DCMD system. (**B**) (**i**) Downstream alterations of CPC for different inlet feed concentrations of a DCMD system; (**B**) (**ii**) Alteration of maximum CPC with varying inlet feed concentrations of a DCMD system. (**C**) (**i**) Downstream alteration of $\eta_{t}$ for different inlet feed concentrations of a DCMD system; (**C**) (**ii**) Alteration of mean $\eta_{t}$ with varying inlet feed concentrations of a DCMD system. (**D**) (**i**) Downstream alteration of TPC for different inlet feed concentrations of a DCMD system. (**D**) (**ii**) Alteration of mean value of TPC with varying inlet feed concentration.

Figure 10A(i)–D(i) show the downstream alterations of water flux, CPC, thermal efficiency, and TPC for different feed concentrations. The decrease in water flux with increase of $C_{f}\;$ occurs due to a decrease in vapour pressure difference. This is because the water vapour pressure is dependent on the minus quadratic function of molality. The water production rate decreases from 2.56 CCM for $C_{f}=0\;g/L$ to 1.85 for $C_{f}=250\;g/L$. Besides, as the convective mass transfer coefficient remains constant with the increase of $C_{f}$, the decrease of J leads to a decrease in CPC from 1.134 for $C_{f}=10\;g/L$ to 1.08 for $C_{f}=250\;g/L$, Figure 10B. The decrease in thermal efficiency occurs from 0.37 for $C_{f}=0\;g/L$ to 0.3 for $C_{f}=250\;g/L$, due to the decrease in ${q^{\prime \prime }}_{fg}$, which occurs due to the decrease in J. It was also shown that TPC increases with $C_{f}$, from 0.36 for $C_{f}=0\;g/L$ to 0.52 for $C_{f}=250\;g/L$. This occurs because the heat transfer occurs with the combination of phase change and conduction heat transfers, which depend on J and membrane temperature difference, $T_{f,m}\left(x\right)-T_{p,m}\left(x\right)$, respectively. With the same bulk temperature difference, $T_{f,b}-T_{p,b}$, the conduction heat transfer part is dominant, due to the decreased water flux and phase change transfer. Therefore, membrane temperature difference, $T_{f,m}\left(x\right)-T_{p,m}\left(x\right)$, and TPC increases. 

### 4.5. Influence of Channel Length

To study the influence of channel length, we modelled a counter-current arrangement of a DCMD system for 100≤L≤350 mm, and all other parameters were maintained as the baseline condition. 

**Figure 11:**
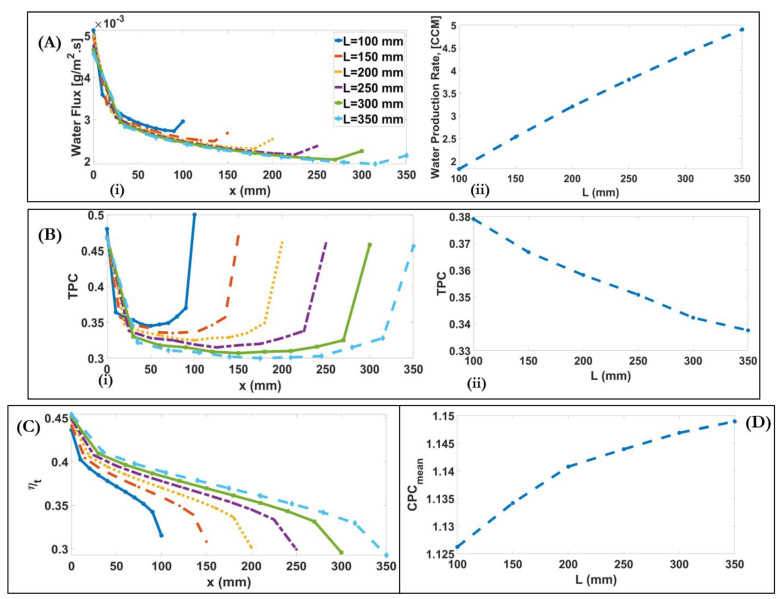
(**A**) (**i**) Downstream alterations of water flux for different channel lengths; (**A**) (**ii**) Alteration of water production rate with varying channel lengths. (**B**) (**i**) Downstream alterations of TPC for different channel lengths; (**B**) (**ii**) Alteration of mean TPC with varying channel lengths. (**C**) Alteration of thermal efficiency with varying channel lengths (**D**)Alteration of CPC with varying channel lengths.

As shown, with the increase of channel length, water flux increases 2.7-fold from 1.82 for 100 mm to 4.9 for 350 mm because the longer channel provides more contact surface to exchange heat between feed and permeate channels; however, water flux decreases at the fixed x, as shown in Figure 11A(i). Due to the decrease of mean membrane temperature difference along the channel, the mean TPC decreases from 0.38 for L=100 mm to 0.34 for L=350 mm as the channel length increases from 100 to 350 mm, Figure 11B(ii). As shown in Figure 11B(i), when the channel length increases, the U-shaped TPC becomes widened. This is because the longer channels provide more heat exchange along the module. Therefore, at the fixed x, the TPC of the shorter channel is higher than the longer one. Figure 11C,D show that thermal efficiency and CPC increase by 8% and 2%, respectively, as the channel length increases from 100 to 350 mm. 

### 4.6. Commercial Membranes 

To analyse the effect of membrane characteristics, 22 different commercially available membranes were explored (Table 2).

**Figure 12:**
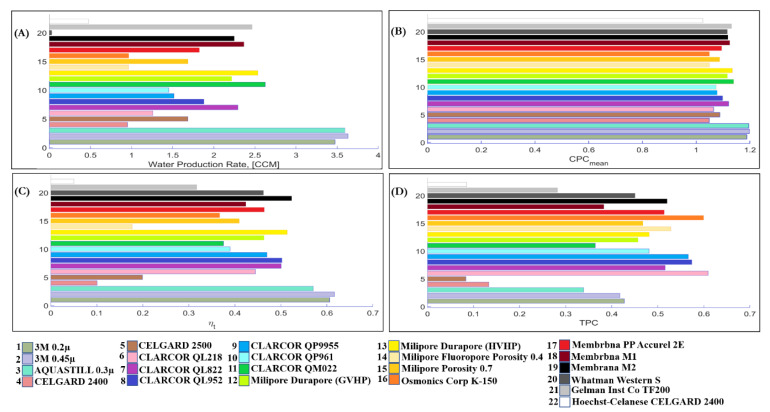
(**A**) Water production rate, (**B**) Maximum of CPC, (**C**) Thermal efficiency $\eta_{t}$, and (**D**) TPC for the 22 commercially available membranes listed in Table 2.

A comparison of the 3M 0.2 µm membrane with 3M 0.45 µm (the only difference is the nominal pore size) showed that different nominal pore size led to a minor change in membrane temperature and vapour pressure difference. It was shown that, with the increase of nominal pore size, from 0.59 µm for 3M 0.2 µm membrane, to 0.79 µm for 3M 0.45 µm, the water flux, CPC, and thermal efficiency increases by 3%, 1.5%, and 1%, respectively (Figure 12A), however TPC slightly decreases 1.5%. This occurs mainly due to the effect of increase of water production rate (Figure 12B–D). 

A comparison of Milipore Porosity 0.4 with Milipore Porosity 0.7 membranes, which have different membrane porosity, 0.4 and 0.7, respectively, showed that, as porosity increases, the water production rate, CPC, and thermal efficiency increases; however, TPC decreases. This is due to the increase in water flux, which occurs due to the increase in the void of membrane. 

Membrana PP Accurel 2E and Membrana M1 were compared to analyze the effect of membrane thickness. As the membrane thickness increases, from 91 µm to 163 µm, TPC increases by 25% (Figure 12D), the water production rate decreases by 22% (Figure 12A), inversely dependent on the membrane thickness, and consequently CPC decreases by 3% (Figure 12B). The increased membrane thickness decreases the water flux and phase change heat transfer, and increases conduction heat transfer, and consequently thermal efficiency by 4%. This is explained by the dominant effect of membrane temperature (Figure 12C).

Figure 12 also shows that it is crucial to prioritize what is expected to obtain from the DCMD system. System performance metrics must be weighed before selecting the commercially available membrane. Concentration polarisation phenomena is related to water flux. Therefore, if membrane fouling is matter of concern, we should make a trade-off between water flux and concentration polarisation. For example, if the water production rate and thermal efficiency are more economically preferable than replacing fouled membranes, membranes manufactured by 3M and AQUASTILL are of great interest, as shown in Figure 12. 

In summary, as can be seen from Table 3, the feed temperature had the most significant effect on water flux and thermal efficiency compared to the other operating conditions. The increased feed temperature enhanced the water flux and thermal efficiency; however, it caused more concentration and temperature polarisation. In contrast, the increased inlet velocity was found to provide increased water flux and reduced temperature and concertation polarisation as well. It was also found that the membrane properties, especially thickness and porosity, can affect the DCMD performance significantly.

## 5. Conclusions

By improving the conventional model that uses one-dimensional and considering downstream alterations, this study performed an extensive and comprehensive analysis of DCMD system performance. Most of the modelling studies that explored DCMD process have limited their investigations to simple correlations to model water flux, minimal discussion on concentration polarisation, broad analysis of different effective parameters, and to few commercially available membranes. Applications of the model showed advantages over the conventional modelling approach, such as capturing downstream alterations along the module, extensive discussion on concentration polarisation effects on different effective parameters of DCMD process, and more accurate water flux values. 

The improved model was in a good agreement with the experimental results. With the increase of feed temperature from 40 °C to 80  °C, the water flux, CPC, and thermal efficiency increased 10-fold, 0.34%, and 0.27%, respectively, and TPC decreased 0.48%. In addition, the ratio of water flux inlet value to outlet value significantly increased by 1.77-fold. By increasing Reynolds number from 80 to 1600, the water flux and TPC enhanced 2.3-fold and 21%, respectively, and the alteration of water flux along the membrane and CPC decreased by 1.74-fold and 2%. By increasing the inlet feed concentration from 0 g/L to 250 g/L, water flux, CPC, and thermal efficiency decreased by 26%, 5%, and 7%; however, TPC increased by 30%. By increasing the length of membrane from 100 mm to 350 mm, the water flux and CPC increased 2.7-fold and 2%; however, TPC decreased by 10%. 

Analysing different aspects of membrane characteristics, it was shown that by increasing the thickness from 91 µm to 163 µm, water flux and CPC decreased by 22% and 3%, and TPC and thermal efficiency increased by 25% and 4%, respectively. By increasing pore size from 0.59 µm to 0.79 µm, the water flux, CPC, and thermal efficiency increased by 3%, 1.5%, and 1%; however, TPC decreased by 1.5%. By increasing porosity from 0.4 to 0.7, the water flux, CPC, and thermal efficiency increased by 73%, 3%, and 23%, respectively; however, it caused a 17% reduction in the TPC.

Our results suggest that increasing velocity and Re number is more valuable than increasing temperature difference in terms of temperature and concentration polarisation phenomena and, therefore, extending our study on turbulence promoter is warranted. However, with the increase of feed temperature, water flux significantly increases. Moreover, the downstream alteration along the membrane is significant. Therefore, it is crucial to study ways to lessen the significant decrease of water flux along the membrane such as by using solar-assisted membrane distillation to localize the heat transfer. Further, the improved model can be used to compare different MD configurations, such as AGMD, which is the focus of our future work.

## Figures and Tables

**Table 1:** Baseline and operating conditions for different parametric studies of a DCMD system.

	Feed Inlet Temperature [°C]	Permeate Inlet Temperature [°C]	Inlet Velocity [m/s]	Feed Concentration [g/L]	Channel Length [mm]	Membrane Type
Baseline condition	60	25	0.05	10	150	CLARCOR, QM0200
Feed inlet temperature study	40–80	25	0.05	10	150	CLARCOR, QM0200
Permeate inlet temperature study	60	20–40	0.05	10	150	CLARCOR, QM0200
Inlet velocity study	60	25	0.01–0.2	10	150	CLARCOR, QM0200
Feed concentration study	60	25	0.05	0–250	150	CLARCOR, QM0200
Channel length study	60	25	0.05	10	100–400	CLARCOR, QM0200
Membrane type study	60	25	0.05	10	150	Table 2

**Table 2:** Membranes selected for this study, and characteristics provided by their manufacturers.

Manufacturer	Model Number	Membrane Type	Nominal Pore Size (µm)	Thickness (µm)	Porosity (%)	Reference
3M	0.2 µm	PP	0.59	110	85	[46]
3M	0.45 µm	PP	0.79	110	85	[46]
AQUASTILL	0.3 µm	PE	0.3	76	85	[46]
CELGARD	2400	PP	0.043	25	41	[46]
CELGARD	2500	PP	0.064	25	55	[46]
CLARCOR	QL218	PTFE	0.2	254–305 (280)	70–85 (75)	[46]
CLARCOR	QL822	PTFE	0.45	127–203 (165)	70–85 (75)	[46]
CLARCOR	QP952	PTFE	0.45	150–300 (223)	70–85 (75)	[46]
CLARCOR	QP9955	PES	0.2	127–305 (216)	70–85 (75)	[46]
CLARCOR	QP961	PES	0.1	76–203 (140)	70–85 (75)	[46]
CLARCOR	QM022	PTFE	0.36	84	0.62	[21]
Milipore	Durapore (GVHP)	PVDF	0.22	125	75	[53]
Milipore	Durapore (HVHP)	PVDF	0.45	140	75	[53]
Milipore	Fluoropore	PTFE	0.22	175	40	[53]
Milipore	Fluoropore	PTFE	0.22	175	70	[53]
Osmonics Corp	k-150	PTFE	0.1	260	75	[53]
Membrana, Germany	PP Accurel 2E	PP	0.2	163	75	[53]
Membrana, Germany	M1	PP	0.2	91	70	[53]
Membrana, Germany	M2	PP	0.45	170	75	[53]
Whatman, Germany	Westran S	PVDF	0.2	121	76	[53]
Gelman Inst Co	TF200	PTFE	0.2	60	60	[53]
Hoechst-Celanese	CELGARD 2400	PP	0.02	25	38	[53]

**Table 3:** Parametric study of DCMD system

Parameter Study		System Performance Metrics				
		Alteration Range	Water Flux (J)	CPC	TPC	Thermal Efficiency $\left(\eta_{t}\right)$
Operating Conditions	Inlet Temperature [ °C ]	40–80	+10-fold	+0.34%	−0.48%	+0.27%
	Inlet velocity [m/s] /Reynolds number	0.01–0.2 80–1600	+2.3-fold	−2%	+21%	−5%
	Inlet Concentration [g/L]	0–250	−0.26%	−5%	+30%	−7%
	Length [mm]	100–350	+2.7-fold	+2%	−10%	+8%
Membrane Properties	Thickness [µm]	M1 with 91 [µm] and Membrana PP Accurel 2E with 163 [µm]	−22%	−3%	+25%	+4%
	Pore size [µm]	3M with 0.59 [µm] and 3M with 0.79 [µm]	+3%	+1.5%	−1.5%	+1%
	Porosity [%]	Milipore with 0.4 and Milipore with 0.7 Porosity	+73%	+3%	−17%	+23%

## Data Availability

Not applicable.

## Supplementary Materials

The following are available online at https://www.mdpi.com/article/10.3390/membranes11050308/s1, Figure S1. (A) Mesh grid generation in ANSYS Workbench-ICEM–Cartesian grid type as a structured grid of DCMD baseline channel. (B) Temperature contour in feed and permeate channels of baseline DCMD system. (C) Velocity contour in feed and permeate channels of counter-current baseline DCMD system. Figure S2. Variation of feed membrane temperature in baseline condition for four different mesh grid size: 40×80, 60×90, 80×120, and 80×100. Figure S3: Downstream variations of feed membrane temperature and vapour pressure difference for different inlet feed temperature, Figure S4: Downstream variations of feed membrane temperature and vapour pressure difference for different inlet permeate temperature. Figure S5. (A) Downstream variations of feed membrane temperature for different inlet velocity of DCMD system. (B) Downstream variations of vapour pressure difference for different inlet velocity of DCMD system. Figure S6. Downstream variations of vapour pressure difference for different inlet feed concentration of DCMD system. Figure S7. (A) Downstream variation of membrane temperature difference between feed-side and permeate-side for different channel length. (B) Variation of membrane temperature difference between feed-side and permeate-side with varying channel length. (C) Variation of membrane vapour pressure difference between feed-side and permeate-side with varying channel length. Figure S8. (A) Mean membrane temperature difference between feed-side and permeate-side for 22 different available commercial membranes listed in Table 2. (B) Mean membrane vapour pressure difference between feed-side and permeate-side for 22 different available commercial membranes listed in Table 2.

## Appendix A

(A1) $$\begin{array}{c} \lambda_{\frac{a}{w}}=\frac{k_{B}T}{\pi {\left[0.5\left(\sigma_{w}+\sigma_{a}\right)\right]}^{2}P_{t}}\cdot \frac{1}{{\left[1+\frac{M_{w}}{M_{a}}\right]}^{0.5}}\\ \sigma_{a}=3.711\times 10^{-10}\\ \sigma_{w}=2.641\times 10^{-10}\\ k_{B}=1.381\times 10^{-23} \end{array}$$

(A2) $$\mathrm{Kn}=\frac{\lambda_{\frac{a}{w}}}{d_{p}}$$

(A3) $$D_{kn}=\frac{4\epsilon }{3\tau }d_{p}\sqrt{\frac{RT}{2\pi M_{w}}}$$

(A4) $$D_{wv-a}=\frac{4.32T^{1.5}\times 10^{-9}}{P_{t}}$$

(A5) $$\alpha =\frac{M_{w}}{M_{a}}$$

(A6) $$p_{sat}=\exp\left(23.5377-\frac{4016.3632}{T-38.6339}\right)$$

(A7) $$a_{w}=1-0.03112M-0.001482M^{2}$$

(A8) $$p_{v}=a_{w}p_{sat}$$

(A9) $$\kappa_{g}=2.72\times 10^{-3}+7.77\times 10^{-5}T$$

(A10) $$\kappa_{m}=\{\begin{array}{l} 5.769\times 10^{-4}T+0.9144\times 10^{-2},\;\;\;\;\;PVDF\\ 5.769\times 10^{-4}T+8.914\times 10^{-2},\;\;\;\;\;\;PTFE\\ 12.5\times 10^{-4}T-23.51\times 10^{-2},\;\;\;\;\;\;\;PP\\ 4.167\times 10^{-4}T+1.452\times 10^{-2},\;\;\;\;\;PES \end{array}$$

(A11) $$\beta =\frac{\kappa_{m}-\kappa_{g}}{\kappa_{m}+2\kappa_{g}}$$

(A12) $$\kappa_{e}=\frac{\kappa_{g}\left(1+2\beta \left(1-\epsilon \right)\right)}{1-\beta \left(1-\epsilon \right)}$$

(A13) $$Nu=4.36+\frac{0.036Re_{h}PrD_{h}/L}{1+0.0011{\left(Re_{h}PrD_{h}/L\right)}^{0.8}}$$

(A14) $$Pr=\frac{c_{P}\mu }{k_{t,{}^{\circ}C}}$$

(A15) $$h_{f,p}=\frac{Nuk_{t,K}}{d_{h}}$$

(A16) $$Re=\frac{\rho ud_{h}}{\mu }$$

(A17) $$\rho =995.3+753.3c-0.008442T^{2}$$

(A18) $$c_{p}=\left(3.112-3.448c+T^{0.003406}\right)\times 10^{3}$$

(A19) $$k_{t,{}^{\circ}C}=0.596-0.448c+0.0006609T$$

(A20) $$\begin{array}{c} k_{t,K}=0.5621+0.00199M+0.000294MT-2.3*10^{-6}MT^{2}+0.00177M^{2}\\ -6.3*10^{-5}TM^{2}+4.5*10^{-7}T^{2}M^{2} \end{array}$$

(A21) $$h_{f}\left(T_{f,b}-T_{f,m}\right)=h_{m}\left(T_{f,m}-T_{p,m}\right)+Jh_{fg}=h_{p}\left(T_{p,m}-T_{p,b}\right)$$

(A22) $$T_{f,m}=\frac{h_{m}\left[T_{p,b}+\frac{h_{f}}{h_{p}}T_{f,b}\right]+h_{f}T_{f,b}-Jh_{fg}}{h_{m}+h_{f}\left[1+\frac{h_{m}}{h_{p}}\right]}$$

(A23) $$C_{f,m}=C_{f,b}\exp\left(\frac{\left(J/\rho_{f}\right)\;}{K_{s}}\right)$$

(A24) $$K_{s}=\frac{ShD_{s}}{d_{h}}$$

(A25) $$Sh=1.86Re^{0.33}Sc^{0.33}{\left(\frac{d_{h}}{L}\right)}^{0.33}$$

(A26) $$D_{s-w}=\left(545.096T\times 10^{-10}+\left(0.086T^{0.5}M^{0.5}-0.162T^{-0.5}M^{0.5}\right)\times 10^{-8}\right)\times 10^{-4}$$

(A27) $$Sc=\frac{\mu }{\rho D_{s}}$$

Additional Equations (A1)–(A27) are shown in the Appendix A [49,50,54,55,56,57].
