# Supplementary material for: An Improved Modelling Approach for the Comprehensive Study of Direct Contact Membrane Distillation
Source: Membranes (Basel). 2021 Apr 22;11(5):308. doi: 10.3390/membranes11050308 (PMC8145701; doi:10.3390/membranes11050308)
Supplement: Supplementary file 1 [file membranes-11-00308-s001.zip › membranes-1175860-supplementary.pdf]

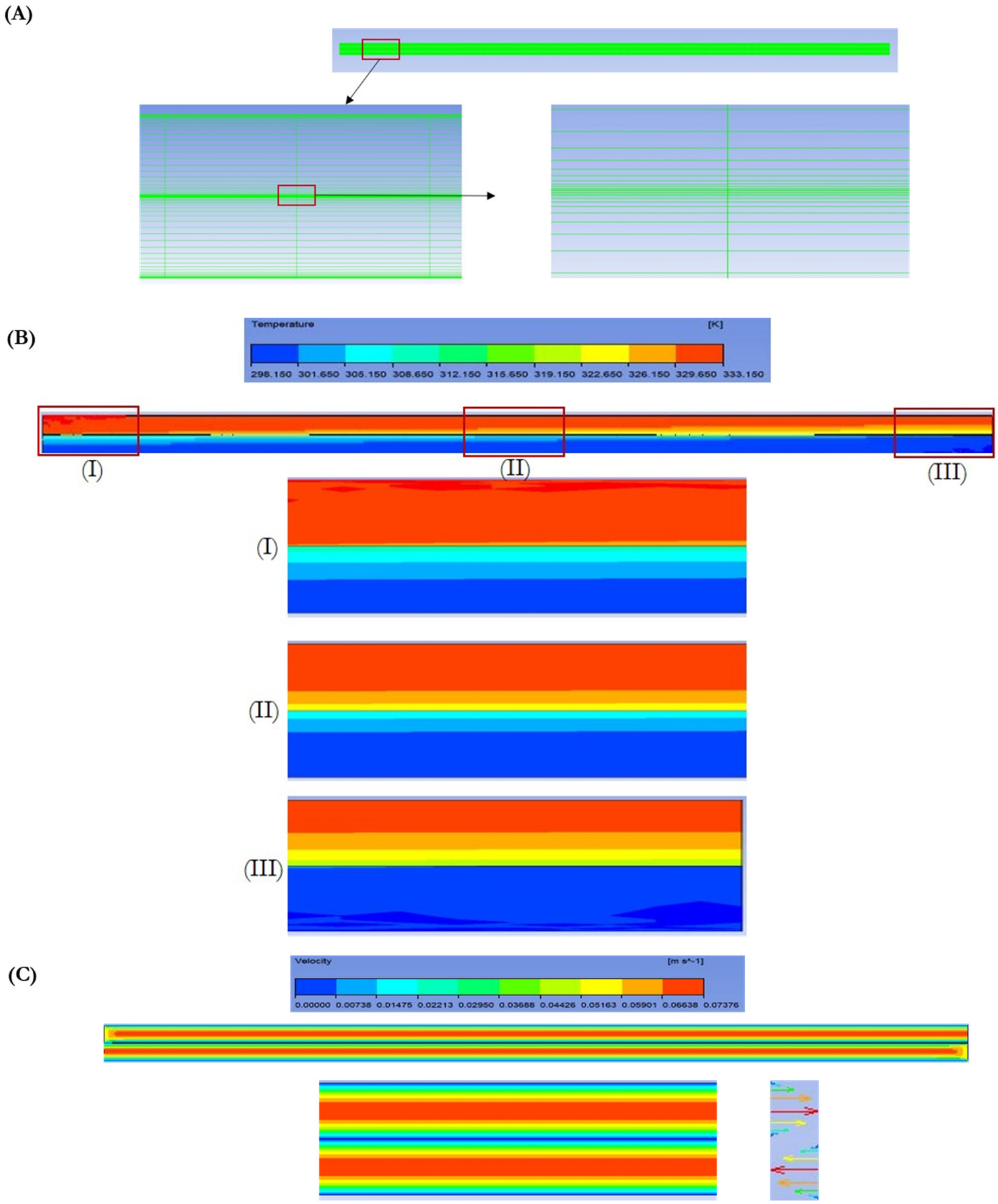

Figure S1. (A) Mesh grid generation in ANSYS Workbench - ICEM – Cartesian grid type as a structured grid of DCMD baseline channel. (B) Temperature contour in feed and permeate channels of baseline DCMD system. (C) Velocity contour in feed and permeate channels of counter-current baseline DCMD system.

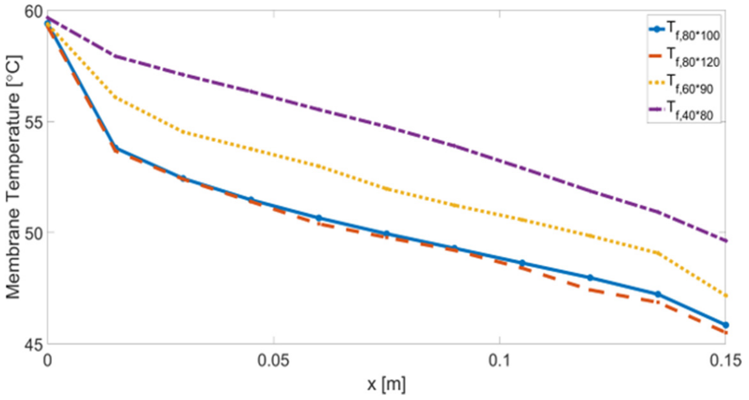

Figure S2. Variation of feed membrane temperature in baseline condition for four different mesh grid size: 40 × 80, 60 × 90, 80 × 120, and 80 × 100

As shown in Figure S1 (A), the DCMD baseline module is discretized using structured quadratic mesh (Cartesian grid type), which captures downstream flow field adjacent to the membrane and both feed and permeate channels. Figures S1 (B) and (C) show the steady state temperature at the inlets, midsections and outlets, and velocity field in a counter-current configuration in a baseline condition as listed in Table 1. After the feed inlet, the velocity on the both feed and permeate side become fully developed along the module, zero at the surface, and developed at the middle.

Figures S3 (A) and (B) show the downstream variation of feed membrane temperature and vapour pressure difference for different feed inlet temperatures, respectively. The membrane temperature difference leads to a vapour pressure difference across the membrane, which decreases in the rightward direction.

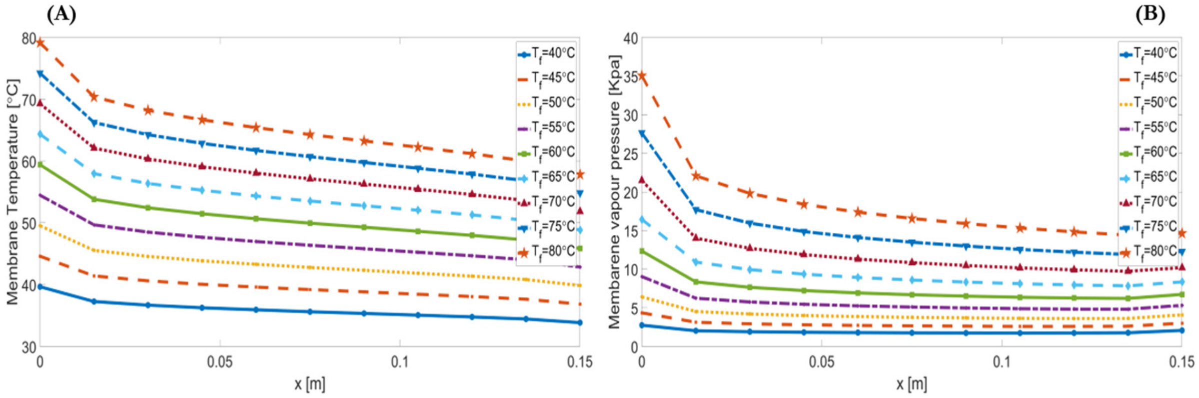

Figure S3. (A) Downstream variations of feed membrane temperature for different inlet feed temperatures of DCMD system. **(B)** Downstream variations of vapour pressure difference for different inlet feed temperatures of DCMD system.

Figures S4 (A) and (B) show the downstream variation of feed membrane temperature and vapour pressure difference for different inlet permeate temperatures, respectively. As the difference between the inlet feed temperature and the inlet permeate temperature decreases with the increase of the inlet permeate temperature, the feed membrane temperature, as shown in Figure S4 (A), for the higher inlet permeate temperature decreases less and more gently. Membrane water vapour pressure difference, which is in a nonlinear fashion dependent on the membrane temperature difference, decreases with the increase of inlet permeate temperature.

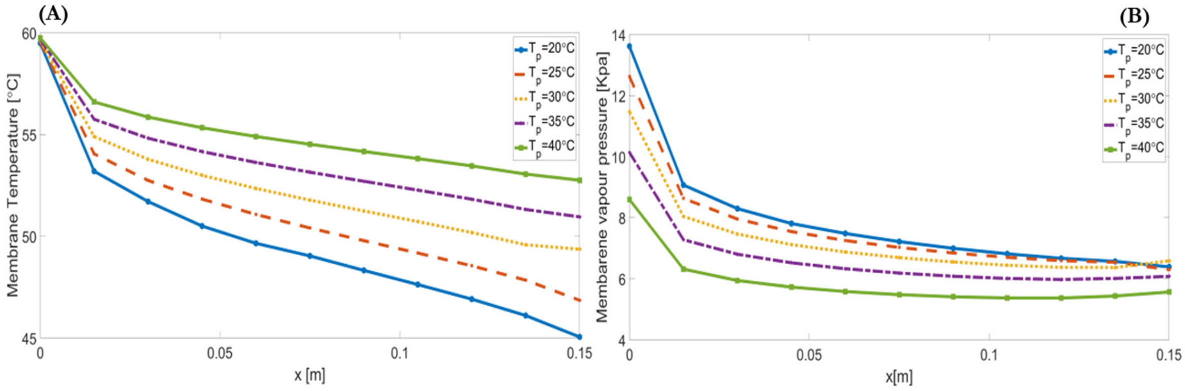

Figure S4. (A) Downstream variations of feed membrane temperature for different inlet permeate temperature of the DCMD system. **(B)** Downstream variations of vapour pressure difference for different inlet permeate temperature of the DCMD system.

Figures S5 (A) and (B) show the downstream variations of feed membrane temperature and vapour pressure difference for different inlet velocity, respectively. As shown in Figure S5 (A), the feed membrane temperature along the membrane decreases. However, with the increase of inlet velocity, the decrease rate of the membrane feed temperature is decreased. This is because the higher inlet velocity leads to a more uniform temperature field in the feed and permeate channels; therefore, the vapour pressure difference increases with the increase of membrane temperature difference, Figure S5 (B).

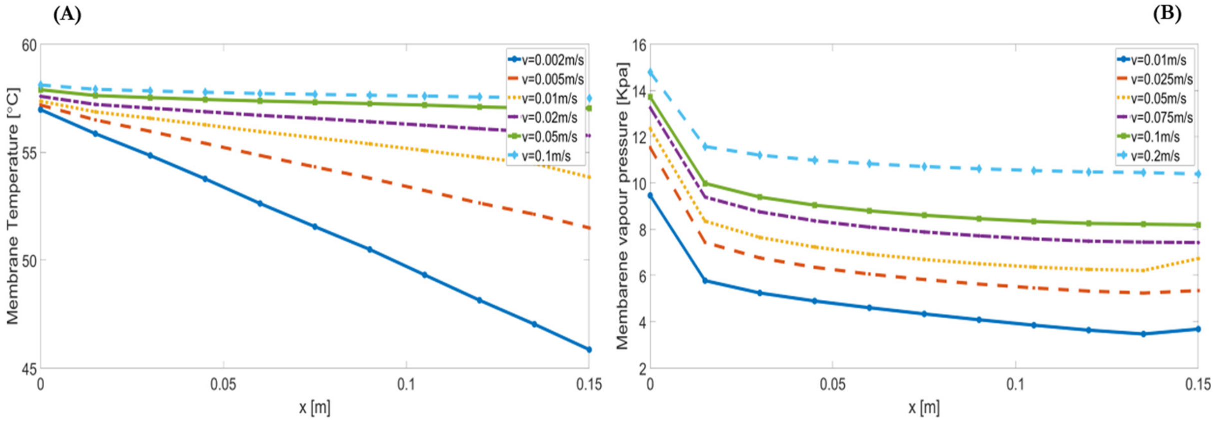

Figure S5. (A) Downstream variations of feed membrane temperature for different inlet velocity of DCMD system. **(B)** Downstream variations of vapour pressure difference for different inlet velocity of DCMD system.

Figure S6 shows the downstream variation of vapour pressure difference along the membrane for different inlet feed concentrations. As the temperature throughout the membrane decreases, the downstream vapour pressure difference decreases. As illustrated, with the increase of feed concentration, water activity of NaCl solution, which is dependent on molality, decreases and, therefore, vapour pressure difference decreases with the increase of the feed concentration.

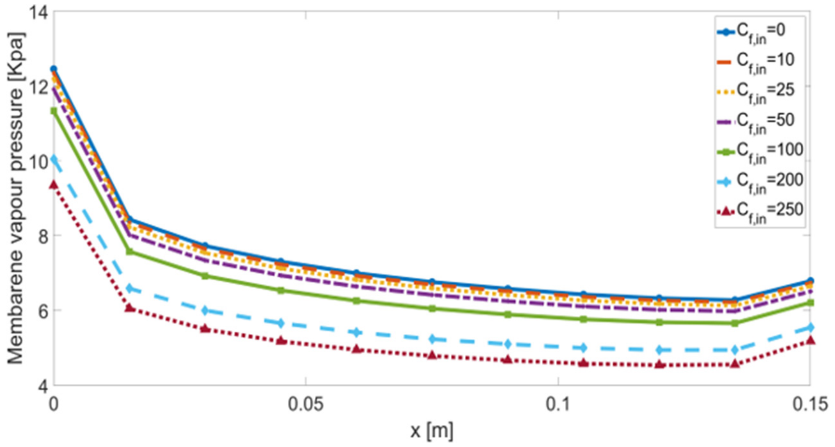

Figure S6. Downstream variations of vapour pressure difference for different inlet feed concentration of DCMD system.

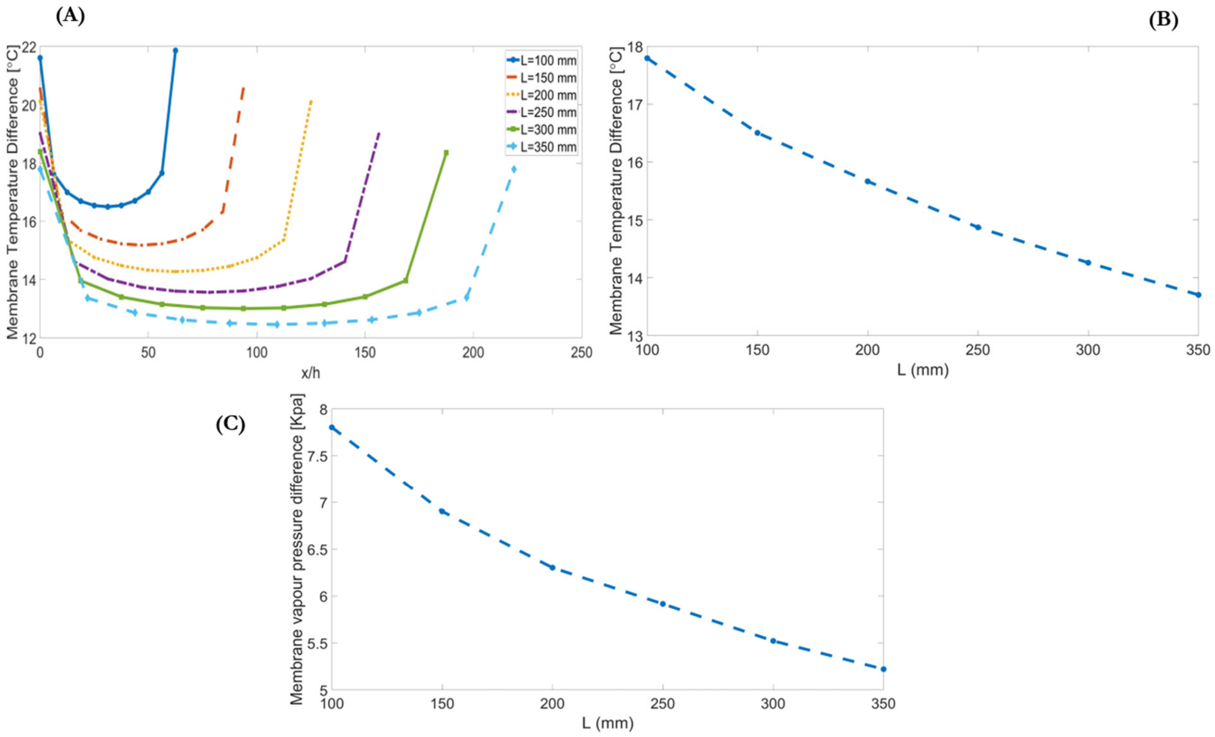

Figure S7. (A) Downstream variation of membrane temperature difference between feed-side and permeate-side for different channel length. **(B)** Variation of membrane temperature difference between feed-side and permeate-side with varying channel length. **(C)** Variation of membrane vapour pressure difference between feed-side and permeate-side with varying channel length.

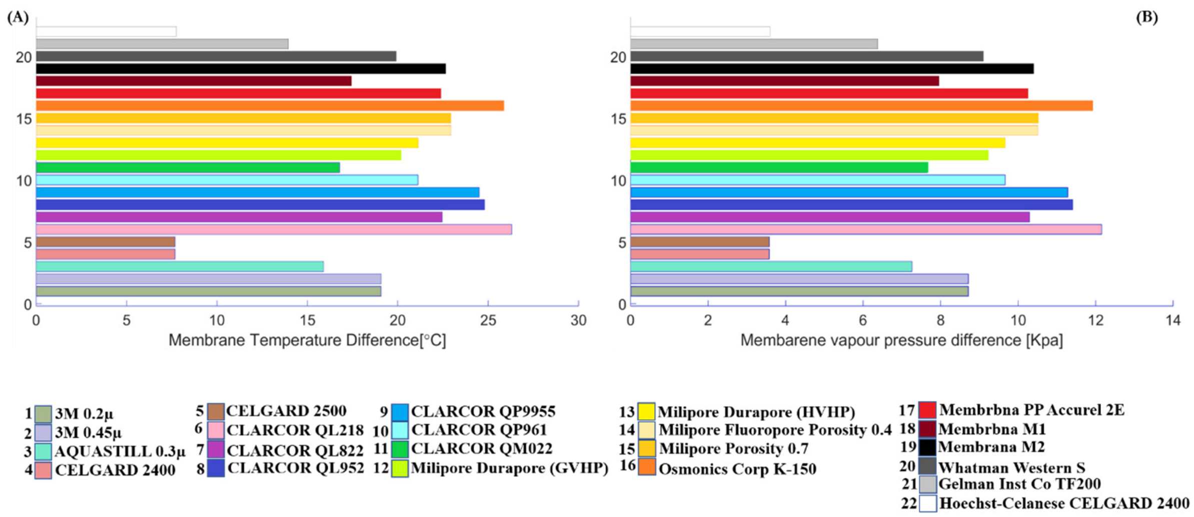

Figure S8. (A) Mean membrane temperature difference between feed-side and permeate-side for 22 different available commercial membranes listed in Table 2. **(B)** Mean membrane vapour pressure difference between feed-side and permeate-side for 22 different available commercial membranes listed in Table 2.
